# The Effect of the Apollo Neuro Device on Anxiety Among Participants who Underwent Ketamine Assisted Therapy

**DOI:** 10.1192/j.eurpsy.2024.875

**Published:** 2024-08-27

**Authors:** V. Tsang

**Affiliations:** UBC, Vancouver, Canada

## Abstract

**Introduction:**

The study aimed to evaluate the effectiveness of a device called “Apollo” in reducing anxiety, as compared to a control group. Participants were divided into two groups: the intervention group (receiving the “Apollo” device) and the control group (receiving no intervention).

**Objectives:**

The primary outcome measure was the change in Generalized Anxiety Disorder 7-item (GAD-7) scores, calculated as the difference between post-GAD-7 and pre-GAD-7 scores.

**Methods:**

Participants were recruited from two different cohorts, with the intervention group derived from the “Apollo” dataset and the control group derived from the “KaT Cohort 9” dataset. Matching was performed based on Age, Sex, and pre-GAD-7 scores to create comparable groups.

For those with full datasets, a total of 4 (out of 5) participants from the “Apollo” group were matched with 15 (out of 45) participants from the “Control” group, based on the selected criteria. Data cleaning was performed to handle missing values and non-numeric entries. Propensity score matching was used to match participants from the “Apollo” and “Control” groups based on Age, Sex, and pre-GAD-7 scores. An independent samples t-test was conducted to compare the mean change in GAD-7 scores between the two groups. Since propensity score matching requires complete data on matching factors (age, sex, pre-GAD-7), those without full datasets were excluded.

**Results:**

The median change in GAD-7 scores in the “Apollo” group was −8.5, indicating a median reduction in anxiety symptoms.The independent samples t-test revealed no statistically significant difference in the change in GAD-7 scores between the “Apollo” and “Control” groups (t = -0.889, p = 0.387). Therefore, the study so far did cannot conclude a significant difference.

**Conclusions:**

Ketamine assisted therapy remains a promising way to decrease anxiety among patients with generalized anxiety disorder and elevated GAD-7 scores. Ways to potentially improve these results are increasing the number of Apollo patients and having more balanced numbers between groups.

**Disclosure of Interest:**

None Declared

